# Prognostic value of CRP–albumin–lymphocyte index in patients with intrahepatic cholangiocarcinoma after radical resection

**DOI:** 10.3389/fmed.2025.1543665

**Published:** 2025-03-06

**Authors:** Shi-Qian Lang, Jun-Jie Kong, Guang-Bing Li, Jun Liu

**Affiliations:** Department of Liver Transplantation and Hepatobiliary Surgery, Shandong Provincial Hospital Affiliated to Shandong First Medical University, Jinan, China

**Keywords:** intrahepatic cholangiocarcinoma, inflammation, nutrition, immunity, prognosis, nomogram

## Abstract

**Purpose:**

The aim of this study is to explore the prognostic value of CRP–Albumin–Lymphocyte (CALLY) index in patients undergoing radical resection of intrahepatic cholangiocarcinoma (ICC).

**Patients and methods:**

Retrospectively collected clinical data of 286 patients with ICC who underwent radical surgery at Shandong Provincial Hospital from July 2010 to July 2021. Univariate and multivariate analyses were used to evaluate the correlation between the CALLY index and overall survival (OS) and recurrence-free survival (RFS), and a nomogram prediction model was established based on the results. The accuracy of the model was evaluated using concordance index (C-index), calibration curves, decision curve analysis (DCA), and the receiver operating characteristic (ROC) curve was used to compare the prognostic value of the nomogram model with the TNM staging system.

**Results:**

The optimal cut-off value of CALLY index was 1.81. In the training set, multifactorial Cox regression analysis showed that CALLY index <1.81 was an independent risk factor for OS and RFS (*p* < 0.05). Compared to neutrophil-to-lymphocyte ratio (NLR), platelet-to-lymphocyte ratio (PLR), systemic immune inflammation index (SII), and modified Glasgow prognostic score (mGPS), CALLY index had a higher area under the curve (AUC). The nomogram established based on the results of multifactorial analysis demonstrated strong efficacy in survival prediction, and the ROC curve showed that the nomogram had a higher prognostic value than TNM staging.

**Conclusion:**

The CALLY index is independently associated with OS and RFS in patients after radical resection of ICC, and the nomogram model based on it shows significantly higher efficacy in predicting the long-term prognosis of patients after radical resection of ICC, and is more accurate than TNM staging.

## Introduction

Intrahepatic cholangiocarcinoma (ICC) is the second most common primary hepatic malignancy after hepatocellular carcinoma (HCC), and it accounts for 10 to 20% of all primary hepatic malignancies. ICC is a relatively rare disease with high malignancy and poor prognosis, and its incidence has been steadily increasing in the past decades. Currently, radical surgical resection remains the mainstay of treatment for ICC, which improves survival in some patients (1, 2). However, the postoperative recurrence rate in ICC patients is as high as 80%, and the 5-year overall survival rate ranges from 20 to 35% (3, 4). Therefore, accurate ICC disease staging is crucial for selecting appropriate treatment methods and predicting patient survival. Some common clinical and pathological features, such as tumor size, tumor number, lymph node metastasis, histological grade, and vascular invasion, can be used to assess the prognosis of patients with ICC (5–7). However, the value of these conventional factors in predicting the prognosis of ICC patients is limited. Recent studies have shown that the occurrence and development of tumors are closely related to immune function, nutritional status, and inflammation levels (8–10), For example: biomarkers such as neutrophil-to-lymphocyte ratio (NLR) (11), platelet-to-lymphocyte ratio (PLR) (12), systemic immune inflammation index (SII) (13), and modified Glasgow prognostic score (mGPS) (14) have been shown to correlate with cancer-specific survival in several cancers. However, these markers have limitations in predicting survival and selecting effective therapeutic strategies, thus better predictive indicators are needed. The CRP–Albumin–Lymphocyte (CALLY) index is a novel immune-nutritional scoring system that combines inflammation, nutrition and immune system status and was first proposed by Iida et al. (15), and has been shown to be associated with prognosis in patients with hepatocellular carcinoma (16), esophageal squamous cell carcinoma (17) and colorectal cancer (18).

In this study, we explored the relationship between CALLY index and prognosis of patients with ICC. And we established a nomogram survival prognostic model based on the CALLY index to predict the survival outcomes of ICC patients treated with curative resection, and compared the survival prediction ability directly with the TNM staging system.

## Materials and methods

### Patient selection

Screening of patients with intrahepatic cholangiocarcinoma who underwent radical liver resection at Shandong Provincial Hospital from July 2010 to July 2021. The inclusion criteria were as follows; (1) patients with histologically confirmed ICC; (2) radical resection; (3) no extrahepatic metastasis; (4) complete baseline laboratory testing information. The exclusion criteria were as follows: (1) patients who had received other treatments (such as transarterial chemoembolization, radiofrequency ablation, or neoadjuvant therapy) prior to surgery; (2) patients with palliative hepatectomy (R1 or R2 resection); (3) patients with a history of other malignant tumors; (4) patients with missing clinical or follow-up data. The included patients were randomized in a 7:3 ratio into a training set (*n* = 200) and a validation set (*n* = 86).

### Data collection and definition of variables

Demographic data include: age, gender, height, weight, smoking status, alcohol consumption and presence of diabetes. Preoperative evaluation of hematologic parameters include: complete blood cell count, liver function tests, serum carbohydrate antigen 19–9 (CA19-9), carcinoembryonic antigen (CEA), and carbohydrate antigen 125 (CA125). Postoperative pathological features include: tumor size, tumor number, peripheral organ invasion, neurological invasion, satellite nodules, histological grade, lymph node metastasis, and TNM staging (following the guidelines of the American Joint Committee on Cancer (AJCC), 8th edition). In addition, several inflammation and nutritional indicators, including NLR, PLR, mGPS and SII were also included in this study. The CALLY index and above inflammatory indicators are calculated as follows (11–13, 15):

$$CALLY-index=\frac{\mathrm{albumin}\;\left(g/\mathrm{dL}\right)\times \mathrm{lymphocyte}\;\left(/\mu L\right)}{\mathrm{CRP}\;\left(\mathrm{mg}/\mathrm{dL}\right)\;x\;104}\;$$

$$NLR=\frac{\mathrm{Neutrocyte}}{\mathrm{Lymphocyte}}\;$$

$$PLR=\frac{\mathrm{Platelet}}{\mathrm{Lymphocyte}}\;$$

$$SII=\frac{\mathrm{Neutrocyte}\times \mathrm{Platelet}}{\mathrm{Lymphocyte}}\;$$

An mGPS of 0 is determined as a CRP level < 10 mg/L and an albumin level > 35 g/L, a score of 1 as a CRP level > 10 mg/L or an albumin level < 35 g/L, and a score of 2 as a CRP level > 10 mg/L and an albumin level < 35 g/L (14).

### Follow-up

Patients were followed up regularly after surgery. Serum CA19-9, CEA, CA125 levels, and liver function were examined during the follow-up period, and CT or MRI was examined every 2–3 months in the first and second years, and every 6 months thereafter until death or loss to follow-up. Tumor recurrence was diagnosed based on elevated serum tumor markers and typical CT or MRI enhanced imaging findings. After diagnosis of tumor recurrence, patients were treated appropriately based on their general condition and the manner in which the tumor recurred. Overall survival (OS) was defined as the duration from the date of surgery to the date of the patient’s death or last follow-up. Recurrence-free survival (RFS) was defined as the duration from the date of surgery to the date of the patient’s disease recurrence or the last follow-up visit.

### Statistical analysis

Continuous variables are expressed as median and interquartile range (IQR) or mean ± standard deviation (SD). Normally distributed variables were tested with Student’s t-test, and variables that did not fit the normal distribution were tested with Mann–Whitney U-test. Categorical variables are shown as numbers and percentages. Differences between groups were compared using the chi-square test or Fisher’s exact probability test. The optimal cutoff values for CALLY index, NLR, PLR, and SII were determined using ROC curves, and the predictive value of each biomarker was compared by comparing the AUC values. Survival curves were generated using the Kaplan–Meier method, and differences between groups were compared using the log-rank test. For the prediction of OS and RFS, hazard ratios (HR) and their 95% confidence intervals (95%CI) were calculated for each variable using univariate and multivariate Cox proportional risk regression models, and variables with *p* < 0.05 in the univariate analyses were included in the multivariate analyses to identify independent risk factors. And prognostic nomograms for survival outcomes (OS and RFS) were created for independent risk factors. The calibration curve was used to assess the calibration of the model (19). The concordance index (C-index) was used to measure the performance and the difference between the predicted and actual outcomes (20). Decision curve analysis (DCA) was used to evaluate the clinical effectiveness of the models (21). The area under the receiver operating characteristic curve (ROC) (AUC) was used to evaluate the discrimination of the model and used to compare its prognostic value with the TNM stage (22).

R software version 4.1.1 was used to plot the nomograms, calibration curves, DCA curves and ROC curves and to calculate the AUC values and C-index. Other statistical analyses were performed using SPSS software (IBM SPSS Statistics, version 22.0; IBM Corporation, Armonk, NY, United States). All tests were two-tailed and a *p* value <0.05 was considered statistically significant.

## Results

### Patient characteristics

A total of 286 patients were included in this study. There were 172 males and 114 females. The median age was 60 years, with a range from 18 to 84 years. The 1-year, 3-year, and 5-year survival rates were 67.1, 30.1, and 17.8%, respectively. The 1-year, 3-year, and 5-year RFS rates were 51.0, 25.5, and 17.0%, respectively.

As shown in Table 1, correlation tests were performed on baseline data and clinicopathologic characteristics of the training set (*n* = 200) and validation set (*n* = 86). The distribution of the two cohorts was relatively balanced (*p* > 0.05).

**Table 1:** The clinicopathological characteristics of ICC patients in the training and validation cohorts.

Variables	Total (*n* = 286)	Training cohort (*n* = 200)	Validation cohort (*n* = 86)	*p*-value
Age, median (IQR), years	60 (52–66)	60 (53–66.8)	58 (51–63.3)	0.053
Gender, *N* (%), male	172 (60.1%)	122 (61.0%)	50 (58.1%)	0.650
BMI, mean ± SD, kg/m2	24.37 ± 3.36	24.17 ± 3.18	24.84 ± 3.71	0.120
Diabetes mellitus, *N* (%)	29 (10.1%)	23 (11.5%)	6 (7.0%)	0.245
Smoking history, *N* (%)	90 (31.5%)	69 (34.5%)	21 (24.4%)	0.092
Drinking history, *N* (%)	85 (29.8%)	64 (32.0%)	21 (24.7%)	0.218
HBV, *N* (%)	72 (25.2%)	48 (24.0%)	24 (27.9%)	0.485
CEA, median (IQR), ng/mL	3.15 (1.91–5.68)	3.26 (1.95–6.51)	2.73 (1.79–4.50)	0.054
CA125, median (IQR), U/ml	19.52 (12.26–38.01)	20.23 (12.43–211.20)	19.46 (11.98–34.45)	0.273
CA199, median (IQR), U/mL	59.20 (17.34–525.25)	61.18 (17.72–511.15)	47.03 (16.90–538.29)	0.673
WBC, mean ± SD, 10^9/L	6.59 ± 2.16	6.76 ± 2.07	6.19 ± 2.30	0.039
L, median (IQR), 10^9/L	1.52 (1.19–1.97)	1.50 (1.18–1.97)	1.55 (1.25–1.98)	0.549
NLR, median (IQR)	2.62 (1.84–3.45)	2.73 (1.84–3.68)	2.42 (1.80–3.18)	0.047
mGPS, *N* (%)				0.345
0	194 (67.8%)	132 (66.0%)	62 (72.1%)	
1	69 (24.1%)	49 (24.5%)	20 (23.3%)	
2	23 (8.0%)	19 (9.5%)	4 (4.7%)	
SII, median (IQR)	561.14 (359.93–851.54)	611.74 (371.97–933.57)	514.74 (330.48–746.11)	0.059
PLR, median (IQR)	140.62 (101.00–187.24)	141.88 (100.84–195.19)	134.77 (101.48–173.03)	0.304
Cally index, median (IQR)	1.08 (0.46–4.50)	1.01 (0.40–4.49)	1.19 (0.60–4.56)	0.235
ALB, median (IQR), g/L	41.40 (38.38–44.43)	40.80 (37.83–43.80)	43.05 (39.70–45.93)	<0.001
TB, median (IQR), μmol/L	15.70 (11.59–20.92)	15.32 (11.57–19.20)	16.80 (11.74–26.31)	0.111
Child-Pugh classification, *N* (%)				0.665
A	249 (87.1%)	173 (86.5%)	76 (88.4%)	
B	37 (12.9%)	27 (13.5%)	10 (11.6%)	
TNM classification, *N* (%)				0.279
IA + B	153 (53.5%)	102 (51.0%)	51 (59.3%)	
II	39 (13.6%)	31 (15.5%)	8 (9.3%)	
IIIA+B	94 (32.9%)	67 (33.5%)	27 (31.4%)	
ASA classification, *N* (%)				0.819
≤II	244 (85.3%)	170 (85.0%)	74 (86.0%)	
III	42 (14.7%)	30 (15.0%)	12 (14.0%)	
Laparoscopic resection, *N* (%)	77 (26.9%)	49 (24.5%)	28 (32.6%)	0.159
Maximum tumor size, median (IQR), cm	5.00 (3.50–7.50)	5.00 (3.50–7.50)	4.80 (3.15–7.00)	0.278
Multiple tumor, *N* (%)	57 (19.9%)	42 (21.0%)	15 (17.4%)	0.490
Adjacent organ invasion, *N* (%)	47 (16.4%)	34 (17.0%)	13 (15.1%)	0.693
Tumor differentiation, *N* (%)				0.110
Poor	108 (37.8%)	79 (39.5%)	29 (33.7%)	
Moderate	171 (59.8%)	114 (57.0%)	57 (66.3%)	
Well	7 (2.4%)	7 (3.5%)	0 (0.0%)	
Perineural invasion, *N* (%)	46 (16.1%)	30 (15.0%)	16 (18.6%)	0.447
Lymph node metastases, *N* (%)	61 (21.3%)	45 (22.5%)	16 (18.6%)	0.461
Satellite lesion, *N* (%)	36 (12.6%)	31 (15.5%)	5 (5.8%)	0.024

Data are presented as *N* (%), median (IQR) or mean ± SD; IQR, interquartile range; SD, standard deviation; ICC, Intrahepatic Cholangiocarcinoma; BMI, Body Mass Index; HBV, Hepatitis B Virus; CEA, Carcinoembryonic Antigen; CA125, Carbohydrate Antigen 125; CA199, Carbohydrate Antigen 19–9; WBC, White Blood Cell; L, Lymphocytes; NLR, Neutrophil-to-Lymphocyte Ratio; mGPS, Modified Glasgow Prognostic Score; SII, Systemic Immune Inflammation Index; PLR, Platelet-to-Lymphocyte Ratio; Cally index, C-reactive Protein-Albumin-Lymphocyte; ALB, Albumin; TB, Total Bilirubin; ASA, American Society of Anesthesiologists.

### Survival analysis for OS

**Figure 1:**
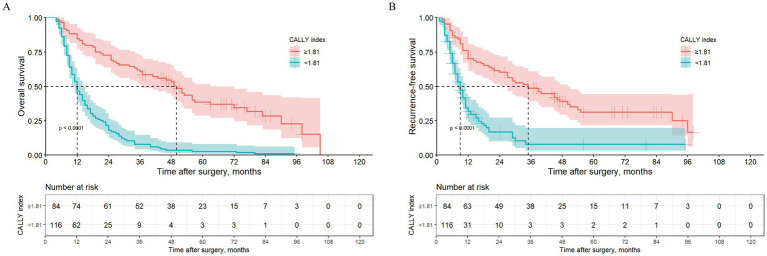
Kaplan–Meier curves comparing OS **(A)** and RFS **(B)** between ICC patients with CALLY-index ≥1.81 and CALLY-index <1.81in the training cohort.

In the training set, Kaplan–Meier survival analysis and log-rank test results showed that patients with high CALLY-index had better OS than those with low CALLY-index. The estimated median OS for patients with high CALLY-index was 50 (95% CI 41.1–58.9) months, while the estimated median OS for patients with low CALLY-index was 12 (95% CI 10.1–13.9) months (*p* < 0.001; Figure 1A).

Univariate analysis suggested that female patients, CEA ≥ 10 ng/mL, CA-199 ≥ 40 U/mL, CA-125 ≥ 40 U/mL, NLR ≥ 2.65, PLR ≥ 167.8, mGPS of 1 or 2, SII ≥ 797.7, CALLY-index ≤1.81, total bilirubin ≥21 umol/L, TNM staging of III, open surgery, maximum tumor diameter ≥ 5 cm, presence of peripheral organ infiltration, tumor hypo-differentiation, presence of lymph node metastasis, and presence of satellite foci were associated with poorer OS (*p* < 0.05). Multifactorial analysis showed that CA-199 ≥ 40 U/mL [hazard ratio (HR): 2.286, 95% confidence interval (95% CI): 1.537–3.398; *p* < 0.001], CALLY-index ≤1.81 (HR: 2.596, 95%CL: 1.614–4.174; *p* < 0.001), and having lymph node metastasis (HR: 2.286, 95% CL: 1.137–4.597; *p* = 0.020), and maximum tumor diameter ≥ 5 cm (HR: 1.873, 95% CL: 1.287–2.726; *p* = 0.001) were independent risk factors associated with OS (Table 2).

**Table 2:** Univariate and multivariate COX regression analysis of overall survival for intrahepatic cholangiocarcinoma (ICC) after radical resection in the training set.

Variables	Comparisons	Univariate analysis		Multivariate analysis	
		Hazard ratio (95% CI)	*p*- value	Hazard ratio (95% CI)	*p*- value
Age, years	≥65 vs. <65	1.137 (0.820–1.576)	0.443		
Gender	Male vs. female	0.726 (0.534–0.988)	**0.042**	1.030 (0.716–1.481)	0.875
BMI, kg/m2	≥25 vs. <25	0.817 (0.599–1.115)	0.203		
CEA, ng/mL	≥10 vs. <10	2.029 (1.401–2.938)	**<0.001**	1.012 (0.668–1.533)	0.956
CA125, U/ml	≥40 vs. <40	2.610 (1.858–3.665)	**<0.001**	1.044 (0.696–1.566)	0.834
CA199, U/ml	≥40 vs. <40	3.648 (2.610–5.098)	**<0.001**	2.286 (1.537–3.398)	**<0.001**
NLR	≥2.65 vs. <2.65	1.699 (1.249–2.310)	**0.001**	0.832 (0.544–1.272)	0.395
mGPS	0				
	1	2.926 (2.044–4.188)	**<0.001**	1.123 (0.731–1.725)	0.597
	2	2.670 (1.628–4.378)	**<0.001**	1.200 (0.657–2.191)	0.554
SII	≥797.7 vs. <797.7	2.161 (1.568–2.980)	**<0.001**	0.878 (0.520–1.481)	0.625
PLR	≥167.8 vs. <167.8	2.035 (1.484–2.790)	**<0.001**	1.506 (0.903–2.510)	0.117
Cally index	≤1.81 vs. >1.81	4.251 (2.999–6.026)	**<0.001**	2.596 (1.614–4.174)	**<0.001**
TB, μmol/L	≥21 vs. <21	1.589 (1.100–2.297)	**0.014**	1.153 (0.749–1.773)	0.518
Child-Pugh classification	B vs. A	1.472 (0.966–2.242)	0.072		
TNM classification	III vs. I or II	3.086 (2.234–4.263)	**<0.001**	2.022 (0.914–4.473)	0.082
ASA classification	III vs. I or II	0.844 (0.551–1.291)	0.434		
Laparoscopic resection	Yes vs. no	0.613 (0.420–0.895)	**0.011**	0.808 (0.529–1.234)	0.324
Maximum tumor size, cm	≥5 vs. <5	2.076 (1.517–2.840)	**<0.001**	1.873 (1.287–2.726)	**0.001**
Multiple tumor	Yes vs. no	1.411 (0.982–2.027)	0.062		
Adjacent organ invasion	Yes vs. no	1.737 (1.183–2.552)	**0.005**	0.570 (0.303–1.071)	0.081
Tumor differentiation	Poor vs. moderate or well	1.542 (1.134–2.098)	**0.006**	1.172 (0.832–1.652)	0.364
Perineural invasion	Yes vs. no	1.488 (0.985–2.249)	0.059		
Lymph node metastases	Yes vs. no	4.671 (3.237–6.739)	**<0.001**	2.286 (1.137–4.597)	**0.020**
Satellite lesion	Yes vs. no	1.905 (1.282–2.830)	**0.001**	1.005 (0.649–1.555)	0.982

Bold text hinted that these variables were statistically significant. CI, confidence interval; BMI, Body Mass Index; CEA, Carcinoembryonic Antigen; CA125, Carbohydrate Antigen 125; CA199, Carbohydrate Antigen 19–9; NLR, Neutrophil-to-Lymphocyte Ratio; mGPS, Modified Glasgow Prognostic Score; SII, Systemic Immune Inflammation Index; PLR, Platelet-to-Lymphocyte Ratio; Cally index, C-reactive Protein-Albumin-Lymphocyte; TB, Total Bilirubin; ASA, American Society of Anesthesiologists.

### Survival analysis for RFS

In the training set, Kaplan–Meier survival analysis and log-rank test results showed that patients with high CALLY-index also had RFS compared to those with low CALLY-index. The estimated median RFS for patients with high CALLY-index was 35 (95% CI 22.9–47.1) months, while the estimated median RFS for patients with low CALLY-index was 9 (95% CI 7.4–10.7) months (*p* < 0.001; Figure 1B).

Univariate analysis suggested that BMI <25Kg/m2, CEA ≥10 ng/mL, CA-199 ≥ 40 U/mL, CA-125 ≥ 40 U/mL, NLR ≥2.65, PLR ≥167.8, mGPS of 1 or 2, SII ≥797.7, CALLY-index ≤1.81, and total bilirubin ≥21umol/L, TNM stage III, maximum tumor diameter ≥ 5 cm, multiple tumors, presence of peripheral organ infiltration, presence of lymph node metastasis, and presence of satellite foci were all associated with poorer RFS (*p* < 0.05). Multifactorial regression analysis showed that CA-199 ≥ 40 U/mL (HR: 2.197, 95%CL: 1.433–3.369; *p* < 0.001), CALLY-index ≤1.81 (HR: 1.698, 95%CL: 1.027–2.807; *p* = 0.039), maximum tumor diameter ≥ 5 cm (HR: 1.519, 95%CL: 1.019–2.263; *p* = 0.040), multiple tumors (HR: 1.751, 95%CL: 1.168–2.624; *p* = 0.007), and the presence of lymph node metastasis (HR: 2.461, 95%CL: 1.179–5.139; *p* = 0.016) were independent risk factors associated with RFS **(**Table 3**)**.

**Table 3:** Univariate and multivariate COX regression analysis of recurrence-free survival for intrahepatic cholangiocarcinoma (ICC) after radical resection in the training set.

Variables	Comparisons	Univariate analysis		Multivariate analysis	
		Hazard ratio (95% CI)	*p*- value	Hazard ratio (95% CI)	*p*- value
Age, years	≥65 vs. <65	1.328 (0.938–1.881)	0.110		
Gender	Male vs. female	0.790 (0.563–1.108)	0.172		
BMI, kg/m2	≥25 vs. <25	0.687 (0.487–0.969)	**0.032**	0.892 (0.614–1.296)	0.549
CEA, ng/mL	≥10 vs. <10	1.643 (1.081–2.495)	**0.020**	0.922 (0.566–1.503)	0.746
CA125, U/ml	≥40 vs. <40	2.054 (1.406–3.000)	**<0.001**	0.757 (0.473–1.211)	0.246
CA199, U/ml	≥40 vs. <40	3.405 (2.366–4.902)	**<0.001**	2.197 (1.433–3.369)	**<0.001**
NLR	≥2.65 vs. <2.65	1.565 (1.121–2.184)	**0.009**	0.838 (0.527–1.332)	0.456
mGPS	0				
	1	2.721 (1.822–4.065)	**<0.001**	1.285 (0.763–2.163)	0.346
	2	2.755 (1.566–4.846)	**<0.001**	1.580 (0.820–3.045)	0.172
SII	≥797.7 vs. <797.7	2.252 (1.581–3.206)	**<0.001**	1.251 (0.689–2.270)	0.462
PLR	≥167.8 vs. <167.8	1.878 (1.330–2.653)	**<0.001**	1.129 (0.666–1.916)	0.652
Cally index	≤1.81 vs. >1.81	2.967 (2.052–4.290)	**<0.001**	1.698 (1.027–2.807)	**0.039**
TB	≥21 vs. <21	1.521 (1.018–2.274)	**0.041**	1.086 (0.675–1.747)	0.733
Child-Pugh classification	B vs. A	1.536 (0.972–2.427)	0.066		
TNM classification	III vs. I or II	2.475 (1.738–3.523)	**<0.001**	1.068 (0.453–2.515)	0.881
ASA classification	III vs. I or II	1.050 (0.671–1.642)	0.831		
Laparoscopic resection	Yes vs. no	0.704 (0.471–1.052)	0.087		
Maximum tumor size, cm	≥5 vs. <5 cm	1.877 (1.335–2.638)	**<0.001**	1.519 (1.019–2.263)	**0.040**
Multiple tumor	Yes vs. no	1.952 (1.343–2.837)	**<0.001**	1.751 (1.168–2.624)	**0.007**
Adjacent organ invasion	Yes vs. no	1.626 (1.065–2.481)	**0.024**	0.796 (0.405–1.565)	0.508
Tumor differentiation	Poor vs. moderate or well	1.270 (0.906–1.781)	0.166		
Perineural invasion	Yes vs. no	1.242 (0.780–1.976)	0.361		
Lymph node metastases	Yes vs. no	3.610 (2.428–5.366)	**<0.001**	2.461 (1.179–5.139)	**0.016**
Satellite lesion	Yes vs. no	2.681 (1.758–4.087)	**<0.001**	1.616 (0.990–2.640)	0.055

Data are presented as *N* (%) or median (IQR); Bold text hinted that these variables were statistically significant. BMI, Body Mass Index; CEA, Carcinoembryonic Antigen; CA125, Carbohydrate Antigen 125; CA199, Carbohydrate Antigen 19–9; NLR, Neutrophil-to-Lymphocyte Ratio; mGPS, Modified Glasgow Prognostic Score; SII, Systemic Immune Inflammation Index; PLR, Platelet-to-Lymphocyte Ratio; Cally index, C-reactive Protein-Albumin-Lymphocyte; TB, Total Bilirubin; ASA, American Society of Anesthesiologists.

### Comparison of the prognostic value of the CALLY index with NLR, PLR, SII, and mGPS

**Figure 2:**
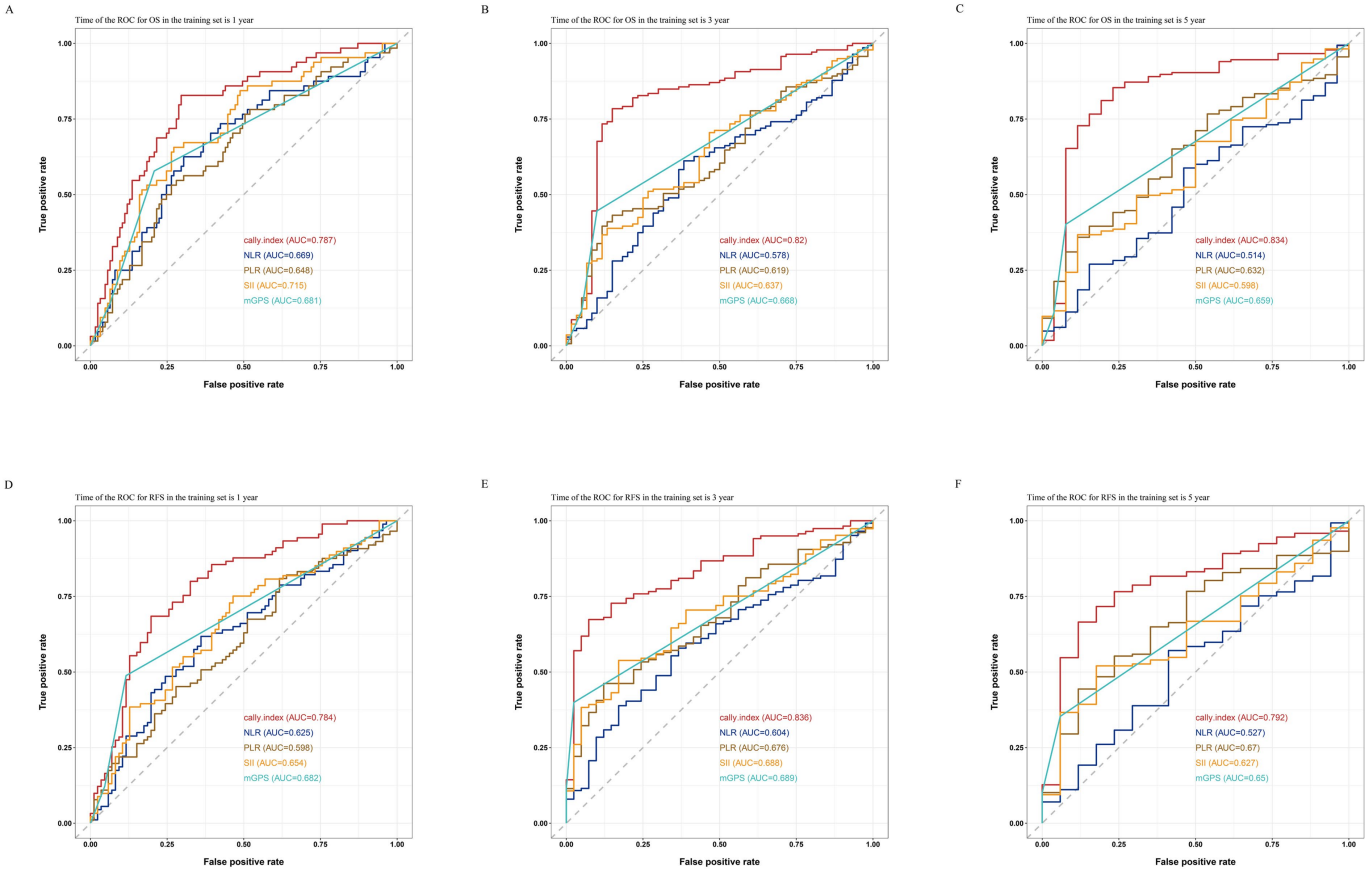
Comparisons of ROC curves for the CALLY-index and the NLR, PLR, SII, and mGPS in predicting 1-year **(A)**, 3-year **(B)**, and 5-year **(C)** OS and 1-year **(D)**, 3-year **(E)**, and 5-year **(F)** RFS in the training set.

As shown in the Figure 2, by comparing the ROC curves for OS and RFS at 1, 3, and 5 years for the CALLY-index with those of NLR, PLR, SII, and mGPS, and obtaining the corresponding AUC values. The results showed that in the training set, in terms of prediction of OS, the AUC values of the CALLY index at 1 year, 3 years, and 5 years were 0.787, 0.820, and 0.834, which were significantly higher than the AUC values of the NLR (0.669, 0.578, and 0.514), the PLR (0.648, 0.619, and 0.632), the SII (0.715, 0.637, 0.598), and mGPS (0.681, 0.668, 0.659); for RFS prediction, the AUC values of the CALLY index at 1 year, 3 years, and 5 years were 0.784, 0.836, and 0.792, respectively, which were also significantly higher than those of NLR (0.625, 0.604, and 0.527), PLR (0.598, 0.676, 0.670), SII (0.654, 0.688, 0.627), and mGPS (0.682, 0.689, 0.650) AUC values. It indicated that CALLY-index possessed higher prognostic value than NLR, PLR, SII and mGPS.

### Nomogram construction and validation

**Figure 3:**
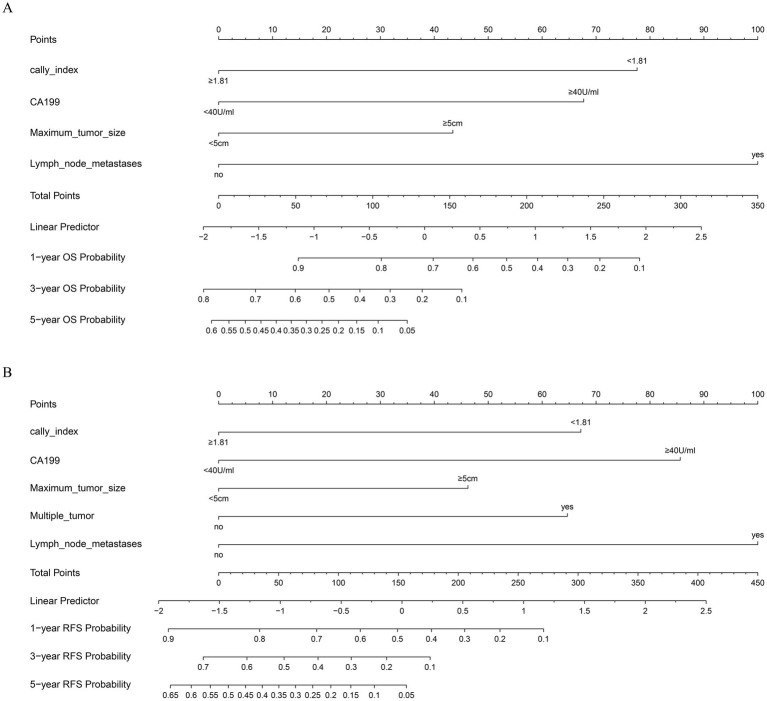
Construction and validation of the nomograms. Nomograms incorporating the CALLY-index and other clinicopathological parameters for OS **(A)** and RFS **(B)** prediction in the training cohort.

**Figure 4:**
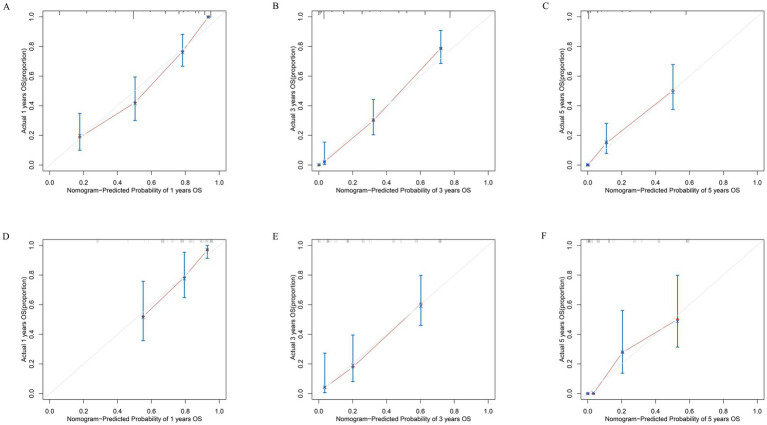
Calibration curves. The calibration curves of the nomograms between predicted and observed 1-year **(A)**, 3-year **(B)**, and 5-year **(C)** OS in the training set and 1-year **(D)**, 3-year **(E)**, and 5-year **(F)** OS in the validation set. The dashed line of 45° represents the perfect prediction of the nomogram.

**Figure 5:**
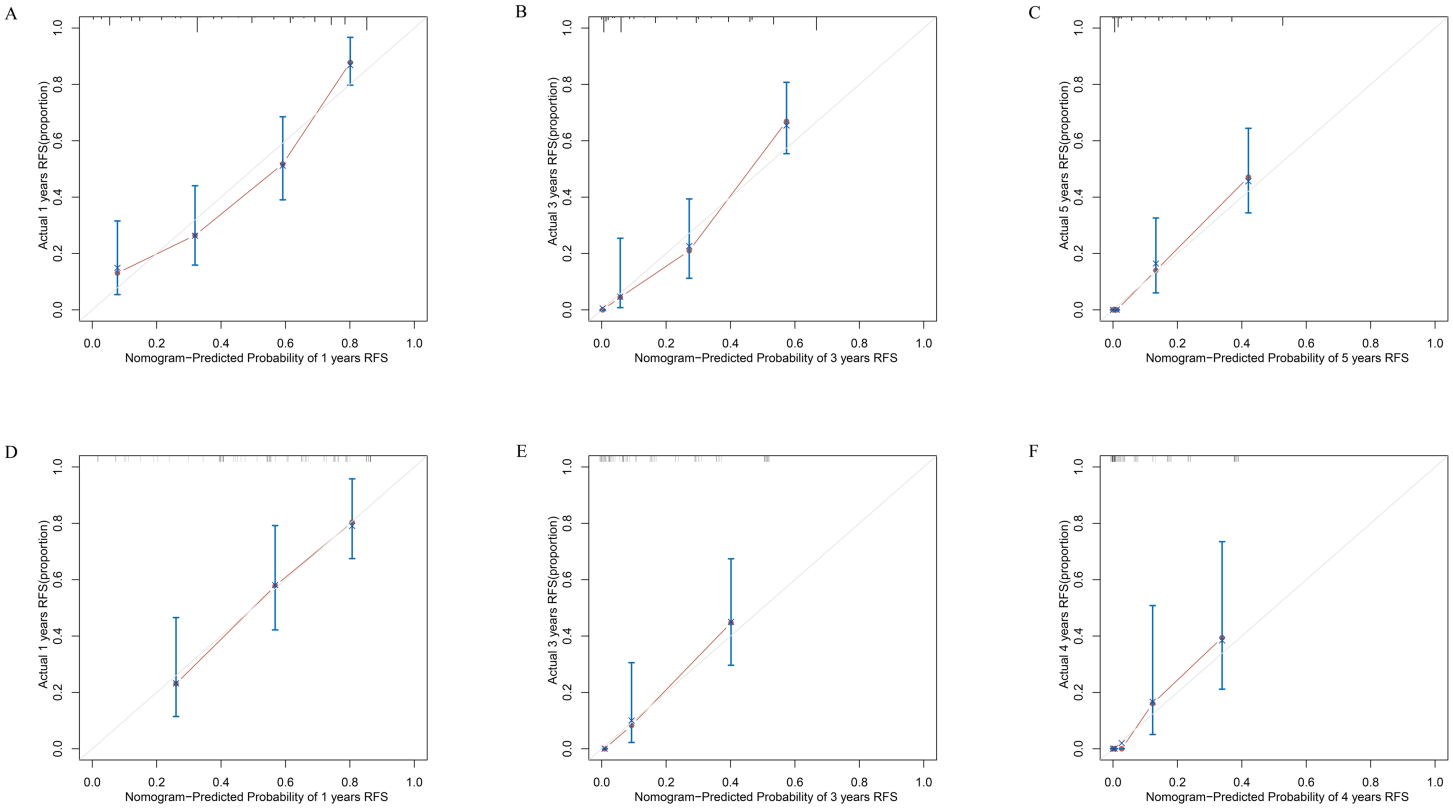
Calibration curves. The calibration curves of the nomograms between predicted and observed 1-year **(A)**, 3-year **(B)**, and 5-year **(C)** RFS in the training set and 1-year **(D)**, 3-year **(E)**, and 4-year **(F)** RFS in the validation set. The dashed line of 45° represents the perfect prediction of the nomogram.

**Figure 6:**
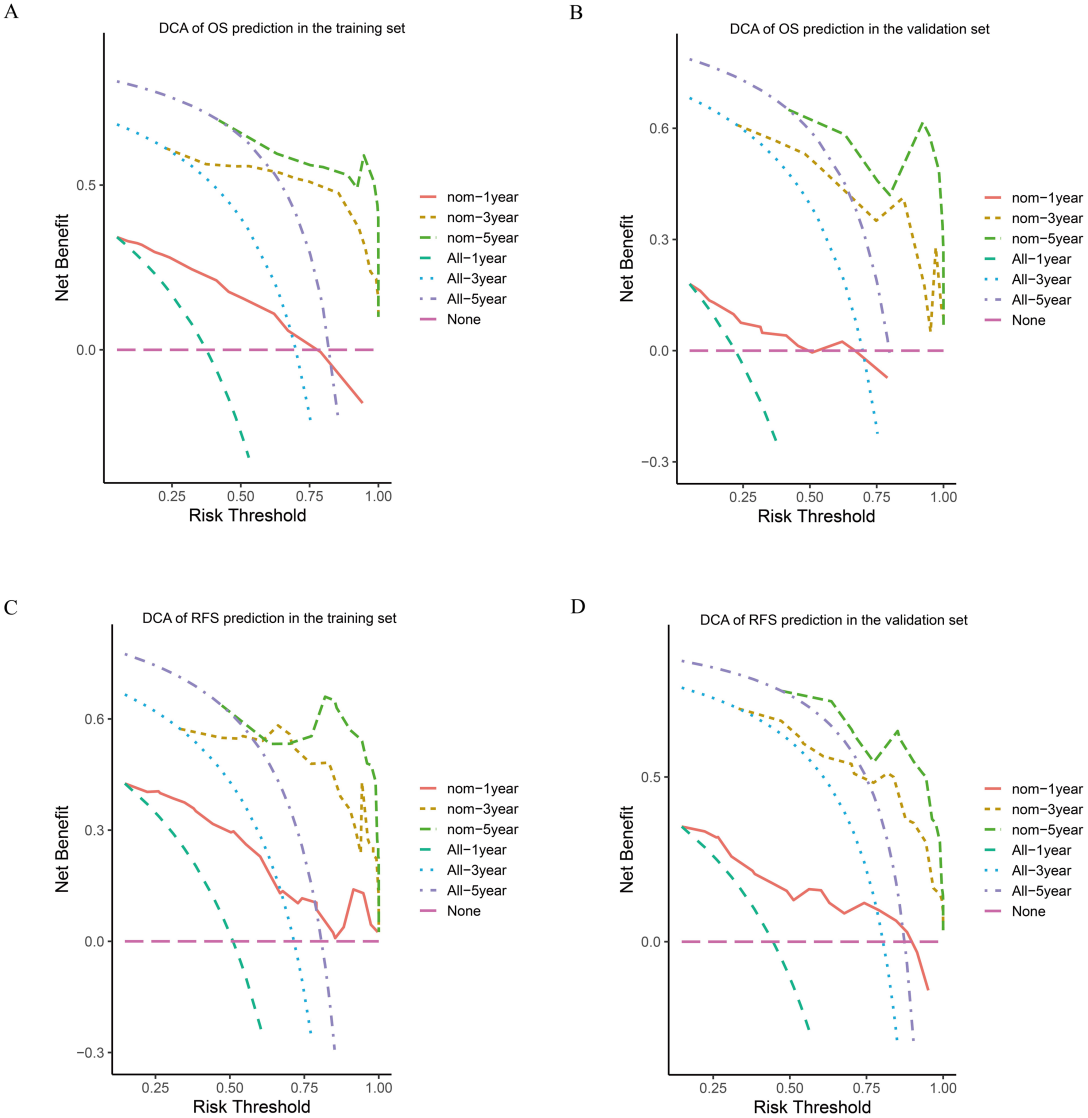
DCA of OS and RFS prediction by the nomograms. The DCA of the nomogram for 1-, 3-, and 5-year OS in the training cohort **(A)** and the validation cohort **(B)**. DCA of the nomogram for 1-, 3-, and 5-year RFS in the training cohort **(C)** and the validation cohort **(D)**.

Based on the results of the multivariate Cox regression analysis, the final nomogram for OS included the CALLY-index, CA-199, maximum tumor size, and lymph node metastasis (Figure 3A). The final nomogram for RFS included the CALLY-index, CA-199, maximum tumor size, multiple tumor, and lymph node metastasis (Figure 3B). It was first validated using C-index. In terms of OS prediction, the C-index for the training and validation sets were 0.795 (95% CI 0.770–0.821) and 0.751 (95% CI 0.696–0.806), respectively. In terms of RFS, the C-index for the training and validation sets were 0.763 (95% CI 0.724–0.802) and 0.756 (95% CI 0.701–0.811), respectively. It suggested that nomogram had good predictive ability. Second, the calibration curves were used to compare the differences between the predicted and actual results. Figure 4 shows that in both the training set (Figures 4A–C) and the validation set (Figures 4D–F), the prediction results of nomogram for OS are in good agreement with the actual results. Figure 5 demonstrates that in the training set (Figures 5A–C) and the validation set (Figures 5D–F), the prediction results of nomogram for RFS also match well with the actual results. Finally, the clinical value of the nomogram was analyzed using DCA curves. The DCA curves of the nomogram for OS (Figures 6A,B) and RFS (Figures 6C,D) were shown in Figure 6. The results indicated that the nomogram prediction model had a good net clinical benefit.

### Comparative performances of the predictive nomograms

**Figure 7:**
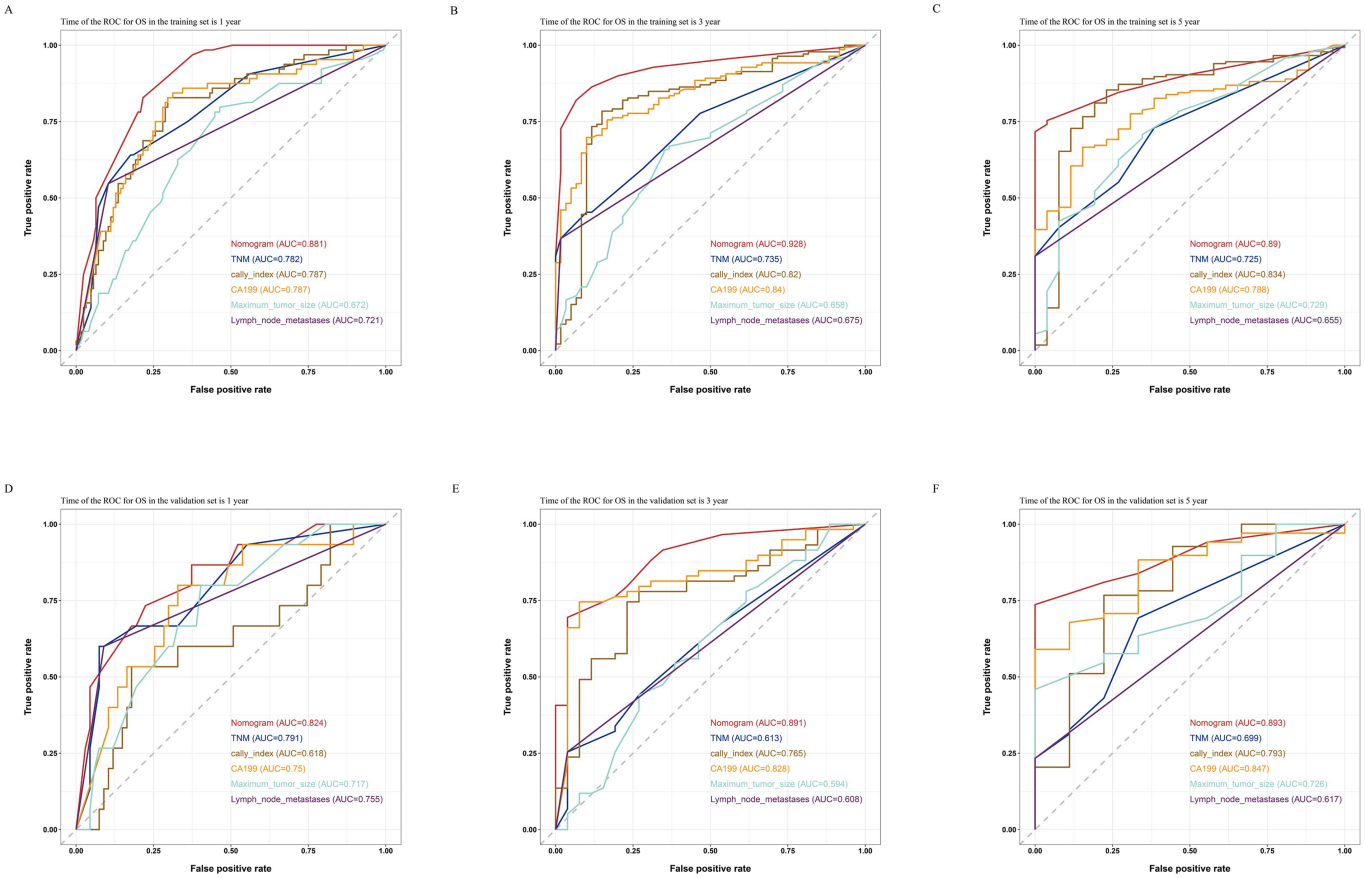
Comparisons of ROC curves of the nomogram, TNM staging and various independent risk factors for 1-year **(A)**, 3-year **(B)**, and 5-year **(C)** OS in the training set and 1-year **(D)**, 3-year **(E)**, and 5-year **(F)** OS in the validation set.

**Figure 8:**
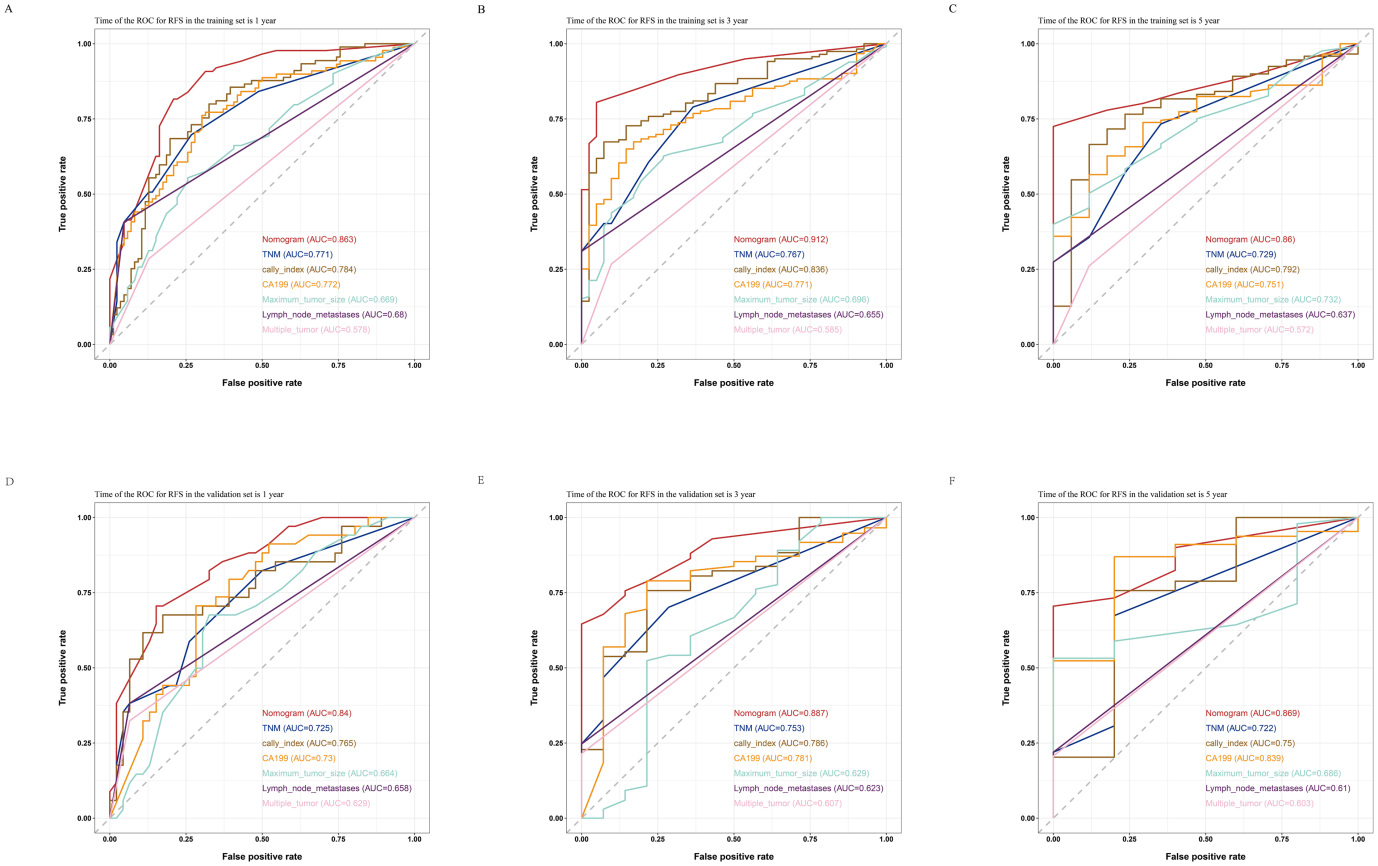
Comparisons of ROC curves of the nomogram, TNM staging and various independent risk factors for 1-year **(A)**, 3-year **(B)**, and 5-year **(C)** RFS in the training set and 1-year **(D)**, 3-year **(E)**, and 5-year **(F)** RFS in the validation set.

By comparing the ROC curves of the nomogram prediction model with the 8th edition AJCC TNM staging and various independent risk factors, we evaluated the predictive capacity. The results showed that in terms of prediction of OS, the nomogram had AUC values of 0.881, 0.928, and 0.890 at 1, 3, and 5 years in the training set (Figures 7A–C) and 0.824, 0.891, and 0.893 in the validation set (Figures 7D–F), which were significantly higher than the AUC values of TNM staging and various independent risk factors. In terms of RFS prediction, the nomogram had AUC values of 0.863, 0.912, and 0.860 at 1, 3, and 5 years in the training set (Figures 8A–C) and 0.840, 0.887, and 0.869 in the validation set (Figures 8D–F), respectively. These values were also significantly higher than the AUC values of TNM staging and various independent risk factors. These results indicate that the nomogram is a prediction model with high predictive ability, surpassing the TNM staging system and various independent risk factors.

## Discussion

In this study, we determined the relationship between the CALLY index and the prognosis of patients after curative resection of ICC. Lower CALLY index was associated with poorer OS and shorter RFS in patients after curative resection of ICC. And its predictive value was stronger than previously proven relevant biomarkers, such as (NLR, PLR, SII, and mGPS). In addition, we also developed a nomogram prediction model including the CALLY index, which showed good predictive ability for OS and RFS, and its predictive ability was stronger than that of the TNM staging commonly used in clinical practice.

In clinical practice, many ICC patients have different postoperative prognoses even though they have the same tumor stage. This may be due to the fact that only the patient’s tumor status is taken into account while ignoring the differences in the patient’s own condition, such as the patient’s preoperative nutritional status and inflammation level, etc. The CALLY index was first proposed by Iida et al. and consists of CRP, serum albumin, and lymphocytes. In clinical practice, CRP is often used to reflect the strength of the inflammatory response in the patient’s body; serum albumin is often used to measure the nutritional status of the patient; and lymphocytes play an important role in the autoimmune response, so the number of lymphocytes can be used to reflect the strength of the patient’s immune function. Therefore, the CALLY index is a comprehensive indicator of the patient’s preoperative inflammation level, nutritional status and immune function.

A large number of studies have now shown that nutritional status, levels of inflammation and immune function are closely related to the occurrence and development of cancer (23, 24). First, in terms of inflammation, the activation of almost all common oncogenes are accompanied by inflammation, and the ensuing hyperinflammatory state can promote tumor maturation (25). In clinical practice, CRP is often used to reflect the level of inflammation in a patient’s body; CRP is an acute-phase protein produced by the liver and regulated by inflammation-related factors such as interleukin-6 and vascular endothelial growth factor. Overexpression of the inflammatory factor IL-6 is present in almost all types of tumors, and it contributes to tumorigenesis and progression by regulating a variety of signaling pathways in cancer (including cell apoptosis, survival, proliferation, invasiveness, and metastasis, as well as the most critical metabolism (26). The fact that CRP, which is regulated by IL-6, rises with IL-6 may be part of the mechanism by which high levels of CRP are associated with poor prognosis in cancer patients. Moreover, there is further research directly indicating that in ICC patients, higher levels of CRP are associated with poor prognosis (27). Secondly, the nutritional status of cancer patients also affects their prognosis directly or indirectly through various ways. On the one hand, when malnutrition exists, the body lacks the required energy and substances and basic metabolic activities are restricted (28). On the other hand, malnourished patients have lower tolerance to surgery, chemotherapy and radiotherapy and have weaker treatment outcomes than patients with better nutritional status (29). Thus, malnutrition and cachexia often portend a poor prognosis of cancer patients (30). In the clinical setting, serum albumin is a readily available and relatively inexpensive indicator for assessing a patient’s nutritional status. The results of many studies have shown that lower levels of serum albumin are associated with poor prognosis in a variety of cancers (31), Including ICC (32). Finally, in terms of immune function, lymphocytes are the main component of the body’s immune system, including T cells, B cells and natural killer cells. These cells can produce antibodies that directly kill viral and tumor cells and can regulate the body’s immune response. Tumor cell clearance depends on cell-mediated immune responses within the body, where T lymphocytes receive antigenic stimuli and differentiate into various effector T cells, and further produce cytotoxic proteins, including perforins and granzymes, and secrete them upon contact with the tumor cells (immune synapses), leading to specific killing without damaging the surrounding normal cells (33). It is now widely accepted that most T-lymphocytes undergo “exhaustion” in response to continuous stimulation by tumor cells, which is characterized by a decrease in effector-related molecules (IFN-*γ*, TNF, and granzymes), as well as a loss of stemness and proliferation potential (34). Thus, lower levels of peripheral blood lymphocyte counts are associated with disruption of immunomodulatory and antitumor functions in patients, and are also predicted to be associated with a poorer prognosis. The above findings show that higher levels of inflammation, poorer nutritional status, and depressed immune function are all associated with a poorer prognosis in patients with ICC, and these validate the results of the present study, in which lower CALLY index tended to encompass one or all of the above three.

A large number of previous studies have shown that some noninvasive biomarkers such as PLR, NLR, SII, and mGPS are prognostic markers for many malignant tumors. In the present study, the CALLY index possessed a higher AUC value compared with previous biomarkers, and the results of multivariate analysis showed that the CALLY index had a stronger predictive ability in terms of OS and RFS in patients after radical resection for ICC. This may be due to the fact that the CALLY index provides a more comprehensive assessment of the patient’s own inflammatory, nutritional, and immune levels than other biomarkers.

Worldwide, TNM staging is the most commonly used staging evaluation system, and it plays a very important role in the treatment of malignant tumors. However, it only takes into account tumor characteristics and ignores the patient’s own basic factors related to cancer prognosis, such as the patient’s own inflammation level, nutritional status and immune level. Therefore, in order to predict the prognosis of ICC patients more individually and accurately, we developed a nomogram including CALLY index, Ca-199, and tumor pathological features. In terms of OS and RFS prediction, the nomogram has a higher c-index and AUC values compared to the TNM staging system, indicating that the nomogram has a higher prognostic value than the TNM staging system. Moreover, the calibration curve showed that the predicted results of nomogram were in good agreement with the actual results, and the DCA curve showed that nomogram had a good net clinical benefit. All of the above results validate the high performance of our established nomogram, and we believe that our nomogram can make up for the limitations of TNM staging and can more accurately predict the prognosis of ICC patients in order to provide a more personalized treatment plan, which will lead to better treatment outcomes for ICC patients.

The strength of this study is that the prognostic role of the CALLY index in ICC patients was validated for the first time with the largest number of participants. Moreover, the CALLY index is inexpensive, easy to use, readily available from routine laboratory test results, and has high prognostic value. Therefore, it may have high practicality in daily clinical practice. However, this study has some limitations. First, this study is a retrospective analysis with a limited sample size, and selection bias is inevitable. In addition, different patients had different postoperative adjuvant treatment regimens, which may have increased the bias of the results of this study. Finally, this study was conducted in a single center and lacked the validation of external data. Therefore, further larger sample size, multicenter studies are still needed to validate our results.

## Conclusion

In summary, the results of this study indicate that the preoperative CALLY index is an independent prognostic factor for patients after radical resection of ICC, and its predictive value is superior to other biological indicators. Moreover, the nomogram established based on the combination of CALLY-index and clinical pathological indicators can accurately predict OS and RFS and its predictive ability is stronger than the TNM staging system. Therefore, we believe that the CALLY index and our established nomogram provide clinicians with a better assessment tool to guide the management and treatment of ICC patients more accurately and individually.

## Data Availability

The raw data supporting the conclusions of this article will be made available by the authors, without undue reservation.
